# 

**Published:** 1856-10

**Authors:** 


### DENTAL REGISTER

### OF THE WEST.

EDITED BY

J. TAFT AND GEO. WATT.

October, 1856.

Published Quarterly at $3 Per Annum.

CINCINNATI:
WRIGHTSON & CO., PRINTERS.
1856.

